# Language acquisition in newborns

**DOI:** 10.7554/eLife.111744

**Published:** 2026-05-28

**Authors:** Marcela Peña

**Affiliations:** 1 Cognitive Neuroscience Laboratory, School of Psychology, Pontificia Universidad Católica de Chile Santiago Chile; 2 National Center for Artificial Intelligence (CENIA FB210017) Macul Chile

**Keywords:** verbal memory, acoustic variability, speaker identity, newborns, functional near-infrared spectrosc, Human

## Abstract

The ability of newborns to distinguish between different voices helps them to establish verbal memories from a very early age.

**Related research article** Visibelli E, Fló A, Baraldi E, Benavides-Varela S. 2025. Verbal episodic processing in newborns. *eLife* **14**:RP109096. doi: 10.7554/eLife.109096.

To be born is not simply to exit the womb; it is to experience a completely new environment, including a wide range of new sights and sounds. For human newborns it also involves entering a world of spoken language.

Inside the womb, humans receive information about their linguistic environment (DeCasper and Fifer, 1980), and this prenatal exposure allows them to begin engaging with continuous speech, and to learn about their native language, from the very first days of life (Mehler et al., 1978). Indeed, newborns show a remarkable ability to process speech units such as phonemes (which allows them to distinguish minimally different words such as *bat* and *cat*; Dehaene-Lambertz and Pena, 2001). They can also process prosody (the use of intonation, stress and rhythm in speech) and recurring patterns of sound (Christophe et al., 2003; Gervain et al., 2008).

Previous work indicates that neonates can store certain properties of the language they hear in memory (Benavides-Varela et al., 2012), but these memories are fragile and can be disrupted when the newborn listens to more speech. How, then, can a newborn learn their native language if it is possible for a new established memory to be overwritten by a newer memory just seconds or minutes later? Now, in eLife, Silvia Benavides-Varela and colleagues at the University Hospital of Padova – including Emma Visibelli and Ana Fló as joint first authors, and Eugenio Baraldi – report the results of experiments which show that the identity of the speaker has a role in newborns forming such memories (Visibelli et al., 2025).

Visibelli et al. tested neonates (aged 0–4 days) while they listened to sequences of pseudowords spoken by an adult woman and man. A pseudoword is a made-up string of sounds (phonemes) that sounds like a real word but is not, and such words are widely used in research into speech processing. Visibelli et al. used pseudowords that had the same consonant-vowel-consonant-vowel structure, such as /mita/ and /dafo/. The pseudowords used in the experiments were also similar in terms of intensity, duration and pitch.

The experiments had three phases: familiarization, interference and test. During the familiarization phase the neonates heard a pseudoword (/mita/, /pelu/, or /voli/) spoken by a female adult for approximately three minutes. Then, during the interference phase, they heard a different pseudoword (/noke/ or /dafo/) spoken by a male adult for approximately three minutes. This was an attempt to disrupt the memory of the word heard during the familiarization phase. Finally, during the test phase, the neonates either heard the original pseudoword spoken for three minutes by the same female adult who spoke it during the familiarization phase (the same-word condition), or a different pseudoword spoken by the same female adult (the novel-word condition).

The aim of the same-word condition was to assess the effect of speaker identity on word memory, whereas the novel-word condition was to determine whether neonates encoded the speaker’s voice rather than the specific word. One group completed the same-word condition first, followed by a nine-minute break, followed by the novel-word condition. A second group completed the novel-word condition first.

To assess memory and recognition of the pseudowords, Visibelli et al. measured brain activity using functional near-infrared spectroscopy (fNIRS). This technique estimates changes in blood oxygenation in the cortex, with increased oxygenation in a particular region of the cortex being taken as a signal of increased neural activity in that region. Previous studies have shown that fNIRS is well suited for studying speech processing in neonates (Wilcox and Biondi, 2015). As in adults, most brain regions involved in speech processing in newborns are located near the Sylvian fissure, particularly the superior temporal gyrus, the inferior frontal gyrus, and the inferior regions of the parietal lobes.

Visibelli et al. found that oxygenation increased during the test phase in the novel-word condition. This increase occurred even though the new word was spoken by the same female adult who had spoken during the familiarization phase. Such a response suggests that the novel word had not been stored in memory, despite the familiarity of the speaker. In contrast, oxygenation decreased during the test phase in the same-word condition. The lack of increased brain activity suggests that the word was recognized as familiar, because it was already encoded in memory. Therefore, the familiarity response of the brain provides an indirect measure of memory formation.

Overall, the findings suggest that speaker identity helps to maintain word representations in the brains of newborns. Thus, even a few days after birth, neonates appear able to encode both the words they hear and the identity of the person who said them. Interestingly, the ability to identify different speakers may provide the continuity needed to track speech over time and across breaks and pauses. Adults typically exploit this ability to segregate streams of speech in noisy environments (Bronkhorst, 2015), and to facilitate speech processing (Nygaard and Pisoni, 1998), and it seems as if neonates are also capable of speech segregation.

Beyond these linguistic findings, this work also highlights the challenges of studying neonates. Newborns show high variability in neural responses due to a variety of factors, such as sleep-wake state, metabolic condition, and anatomical differences. Investigating neural responses therefore requires extensive controls and careful analysis to exclude confounds. There are also factors that cannot be controlled for technical or ethical reasons. Despite all this, by being rigorous and thorough, Visibelli et al. have been able to advance our understanding of early language acquisition and provide methodological insights for developmental neuroscience.
